# Investigations of Phase Transformation in Monocrystalline Silicon at Low Temperatures via Nanoindentation

**DOI:** 10.1038/s41598-017-09411-x

**Published:** 2017-08-17

**Authors:** Shunbo Wang, Hang Liu, Lixia Xu, Xiancheng Du, Dan Zhao, Bo Zhu, Miao Yu, Hongwei Zhao

**Affiliations:** School of Mechanical Science and Engineering, Jilin University, Changchun, 130025 China

## Abstract

Nanoindentations of monocrystalline silicon are conducted to investigate the phase transformation process at a temperature range from 292 K to 210 K. The load-displacement curves are obtained and the residual indents are detected by Raman spectra. MD simulations are also conducted to identify the phase state during nanoindentation. The results show that the low temperature has no influence on the generation of Si-II during loading process of indentation, but the phenomenon of pop-out is inhibited with the temperature decreasing. The probability of pop-out occurrence has a dramatic drop from 260 K to 230 K. Both the generation and propagation of Si-III/XII transformed from Si-II are inhibited by the low temperature, and only *a*-Si was generated as a final phase state.

## Introduction

Monocrystalline silicon is an important semiconductor material in scientific research and industrial applications, like manufacturing of the micro-electro mechanical systems (MEMS), precision optics elements and electronic products. Its mechanical properties have been a research focus for many years^1–4^. Including the hardness, Young’s modulus and stiffness, lots of mechanical properties of silicon have been determined via nanoindentation test, which is a convenient and accurate method by recording penetration load (*P*) and displacement (*h*) during indentation process. Meanwhile, because of the ability of inducing the high hydrostatic pressure and shear stress condition, nanoindentation is also used to investigate the phase transformation of monocrystalline silicon, combined with Raman microspectroscopy^5, 6^, transmission electron microscopy^7, 8^ and *in situ* electrical characterization^9, 10^.

At present, cryogenic engineering, associated with superconductor technology and space exploration, attracts more and more attention and requires high reliable structural functions. The properties and functions of materials are quite different at such extreme condition. But both the mechanical properties and process of the phase transformation of silicon are poorly understood at low temperature. Gridneva *et al*.^11^ reported the hardness of Si and Ge at the temperature down to −200 °C via microhardness tester. At elevated temperatures the hardness became larger, but at low temperatures the hardness was almost temperature independent. Molecular Dynamics (MD) with the temperature ranging from 300 K to 10 K was conducted by Zhao *et al*.^12^. They found that the degree of anisotropy increased with the temperature decreasing and identified the existence of Si-II and Si-XIII during indentation. M. M. O. Khayyat *et al*.^13^ carried out experiments with the temperature ranging from 300 K to 150 K using a microhardness tester, indicating that there was no Si-II formed under the temperature below 200 K. However, the *P-h* curves were not recorded by their research, thus lots of important information from the indentation curves were lost. Johnson *et al*.^14^ used Raman spectra to detect Si-III and Si-XII at different temperatures, over a range of 80–300 K, and found that both the Raman shift and the linewidth depended on temperature strongly. But the transformed phases were only conducted at room temperature, and the phase transformation process under low temperature was not discussed.

This study aims to identify the phase transformation process of monocrystalline silicon through nanoindentation tests at temperatures ranging from 292 K to 210 K. Phase transformation is described and explained through indentation unloading process and MD simulations are performed to identify the phase transformation during nanoindentation loading process.

## Results

### *P-h* curves at different temperatures

**Figure 1:**
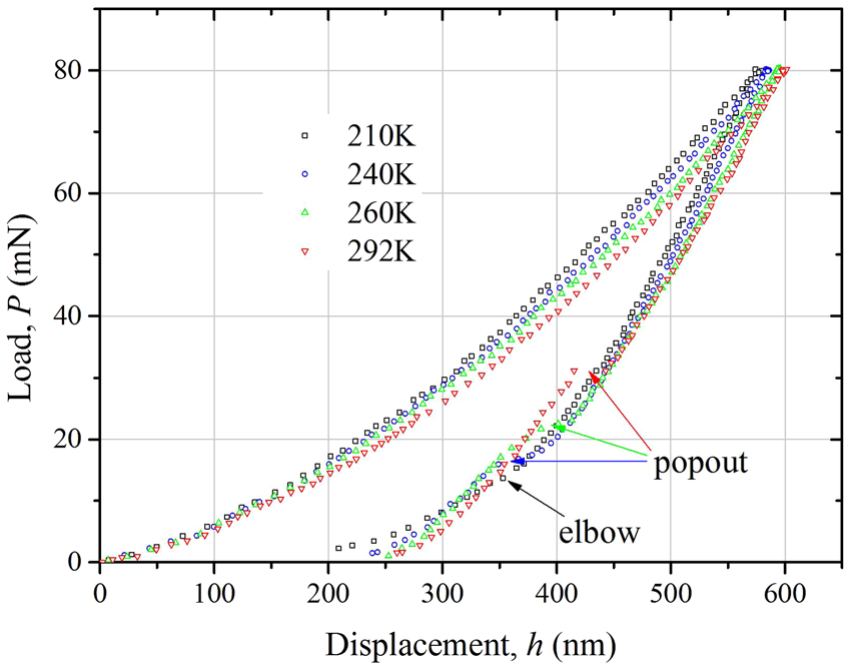
Load-displacement curves for nanoindentation at different temperatures.

It is well accepted that, at room temperature, monocrystalline silicon with the diamond cubic Si-I phase transforms into a much denser metallic Si-II phase (β-Sn phase) during the load process of indentation^15^. But the Si-II phase is unstable and can be transformed into other phases during unloading process. For rapid unloading, the Si-II phase tends to transform into amorphous silicon (*a*-Si), leading to an “elbow” phenomenon. While for slow unloading, most Si-II phase prefers to transform into a mixture of Si-III (bc8) and S-XII (r8) in a quite short time, leading to a discontinuity named as “pop-out”^16^. Fig. 1 shows the *P*-*h* curves obtained via nanoindentation at room temperature 292 K, and low temperature 260 K, 240 K and 210 K. The unloading rate of all the indentations is settled as 1.6 mN/s, which is low enough to guarantee the occurrence of pop-out at room temperature, just as the *P-h* curve conducted at 292 K. It can be seen that at 260 K and 240 K, pop-out also occurs during unloading process. But with the temperature decreasing, pop-out disappears at 210 K, and is substituted by a significant elbow phenomenon.

### Statistical regularity of pop-out events

**Figure 2:**
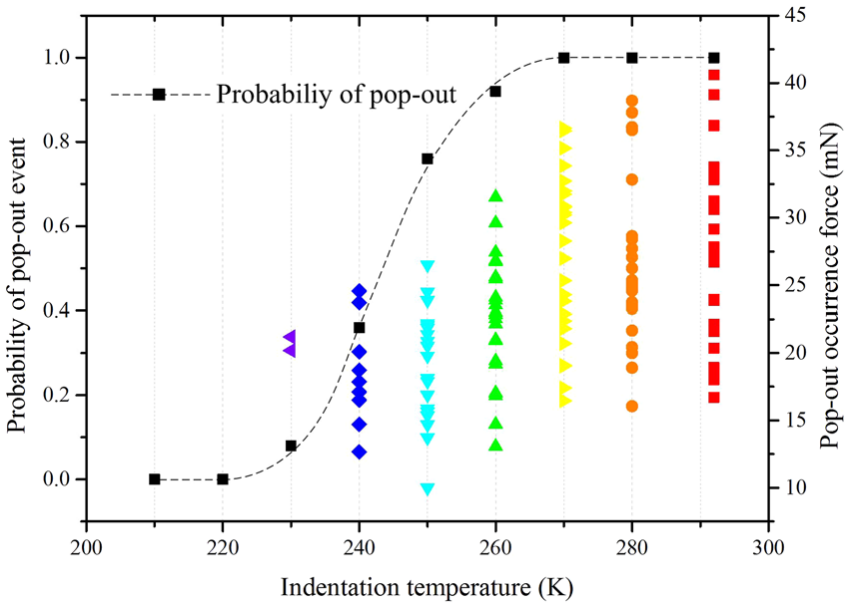
Statistics of pop-out events performed at temperature from 290 K to 210 K of 80 mN indentations.

To investigate the specific regularity of the appearance of pop-out and elbow phenomenon, 25 nanoindentation experiments are conducted at each temperatures from 292 K to 210 K with decrements of 10 K. Both the probability of the occurrence of pop-out and the force at the moment of pop-out occurring are exhibited in Fig. 2. The probability of pop-out declines dramatically when the temperature decreases from 260 K to 230 K, and pop-out disappears below 220 K. All the 225 indentation results show that if pop-out does not occur, the elbow event will exhibit instead. It indicates that the phenomenon of pop-out is inhibited, and elbow is promoted by the low temperature condition. Meanwhile, the critical load for pop-out occurrence at each temperature also decreases significantly with the temperature falling down.

### Typical indentation curves at 240 K

**Figure 3:**
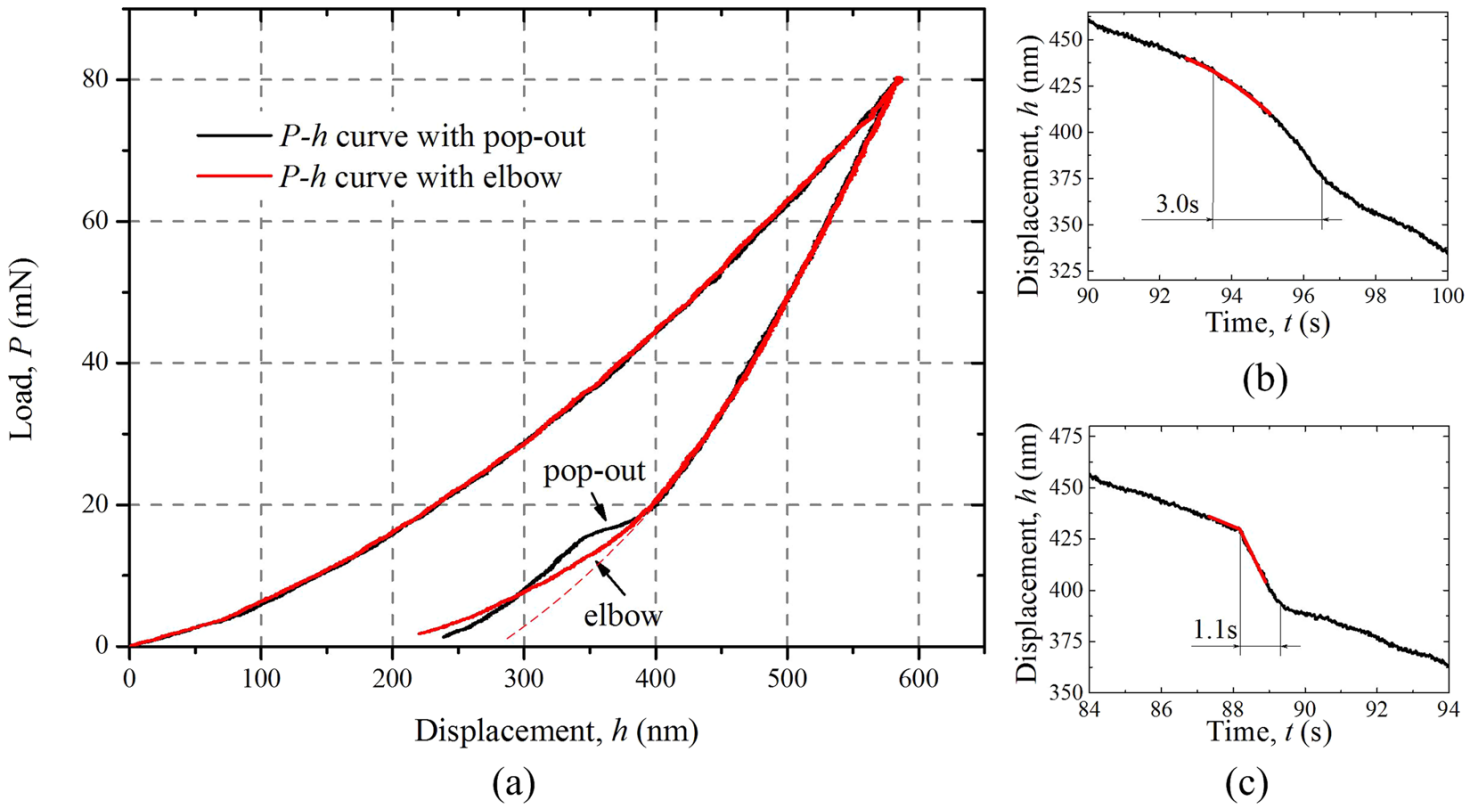
Typical nanoindentation curves. (**a**) Load-displacement curves conducted at 240 K with phenomenon of pop-out and elbow. (**b**) Displacement- time curve with pop-out at 240 K. (**c**) Displacement- time curve with pop-out at 292 K.

From Fig. 2 it can be seen that 240 K is a special temperature that the probability of pop-out occurrence drops fastest and both pop-out and elbow can occur under a similar probability. Two typical *P-h* curves are extracted from 240 K indentations to analyze the phase transformation during unloading process for more details, as shown in Fig. 3a. It can be seen that the two curves coincide very well until the pop-out occurs, indicating that before this moment, the two indentations have a similar mechanical and phase state. The dash line in Fig. 3a shows purely elastic recovery process during unloading process. Both of the curves are separated from the ideal elastic recovery line apparently, as a result of phase transformation with the stress releasing. Additionally, the *h-t* curve corresponding to the pop-out occurring in Fig. 3a is shown in Fig. 3b, whose coordinates are selected to exhibit the pop-out clearly. To make a contrast of pop-out phenomenon at different temperatures, a portion of *h-t* curve conducted at room temperature is shown in Fig. 3c. It can be seen that pop-out at 240 K takes ~3.0 seconds to finish, much longer than the ones occurring under room temperature with ~1.1 seconds. Additionally, there is a significant difference between the two pop-outs during the initial stage (marked with red lines). At 240 K, the beginning of the pop-out is a smooth and gradual process. But at 292 K, the slope of displacement changes suddenly and exhibits as a broken line.

### Raman spectra of residual indents

**Figure 4:**
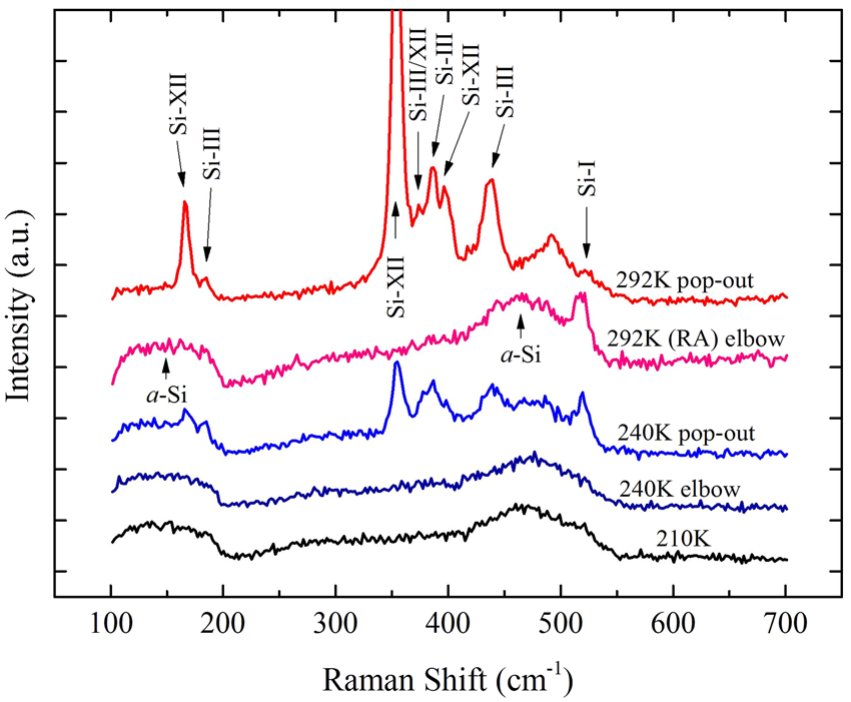
Raman spectra of indents at different temperatures in silicon.

Figure 4 illustrates the Raman spectra results of residual indents conducted at 292 K, 240 K and 210 K with different phenomenons. Shark peaks at ~166, 185, 353, 373.7, 385, 396.3 and 437.5 cm^−1^ shown in the figure indicate the mixture of Si-III/XII phase generated during unloading process^17–19^. Two broad bands at ~150 and 469 cm^−1^ also can be found in the figure, which shows the amorphization of silicon, known as *a*-Si^20^. As shown in the figure, at 292 K, slow unloading rate indentations with pop-out exhibit several peaks, indicating that a mixture of Si-III and Si-XII exist in the residual indents. A rapid unloading indentation (marked as RA) is additionally conducted to make a contrast with the indentations performing pop-out phenomenon. The spectrum of RA shows that only *a*-Si can be found after indentation, where 520 cm^−1^ peak can be ignored representing non-transformed Si-I phase^21^. Meanwhile, two indents conducted at 240 K are carefully detected, which perform pop-out and elbow during unloading process respectively. It can be seen that in the indents with elbow occurring, the structure in the residual area performs an amorphous state, just like “292 K elbow” spectrum. But in the one with pop-out occurring, phase of Si-III/XII generates. However, contrasted with “292 K pop-out” spectrum, the “240 K pop-out” crystal phase peaks are much weaker and broad bands also exist. This indicates that though pop-out occurs at 240 K, not all Si-II phase transforms to Si-III/XII, but some transforming to *a*-Si instead. At 210 K, all the *P-h* curves perform elbows during unloading process, and the spectrum marked with “210 K” is plotted as represent. It is significant that at this temperature only amorphous phase generates, indicating that all the Si-II phase transforms to *a*-Si.

## Discussion

The experiments so far reveal that low temperature has a significant effect on the phase transformation in monocrystalline silicon. According to the results exhibited above, the phenomenon of pop-out is inhibited by the low temperature and *a*-Si tends to be transformed with elbow events. As pop-out exists at 292 K and the probability of its occurrence decreases with a continuous tendency, we can believe that the pop-out occurred at low temperature is also a process that Si-II phase transforms to Si-III/XII. But the elbow event is not sufficient to prove the Si-II existing and transforming to *a*-Si, because it is difficult to identify whether the Si-I or Si-II transforming to *a*-Si and causes the elbow phenomenon. At room temperature *in situ* electrical indentation is an effective method to observe the phase transformation from Si-I to Si-II during loading process of indentation^9, 10^. But the electric characters of Si-II at low temperature are unknown and it is unreliable to evaluate the transformation process via this method.

**Figure 5:**
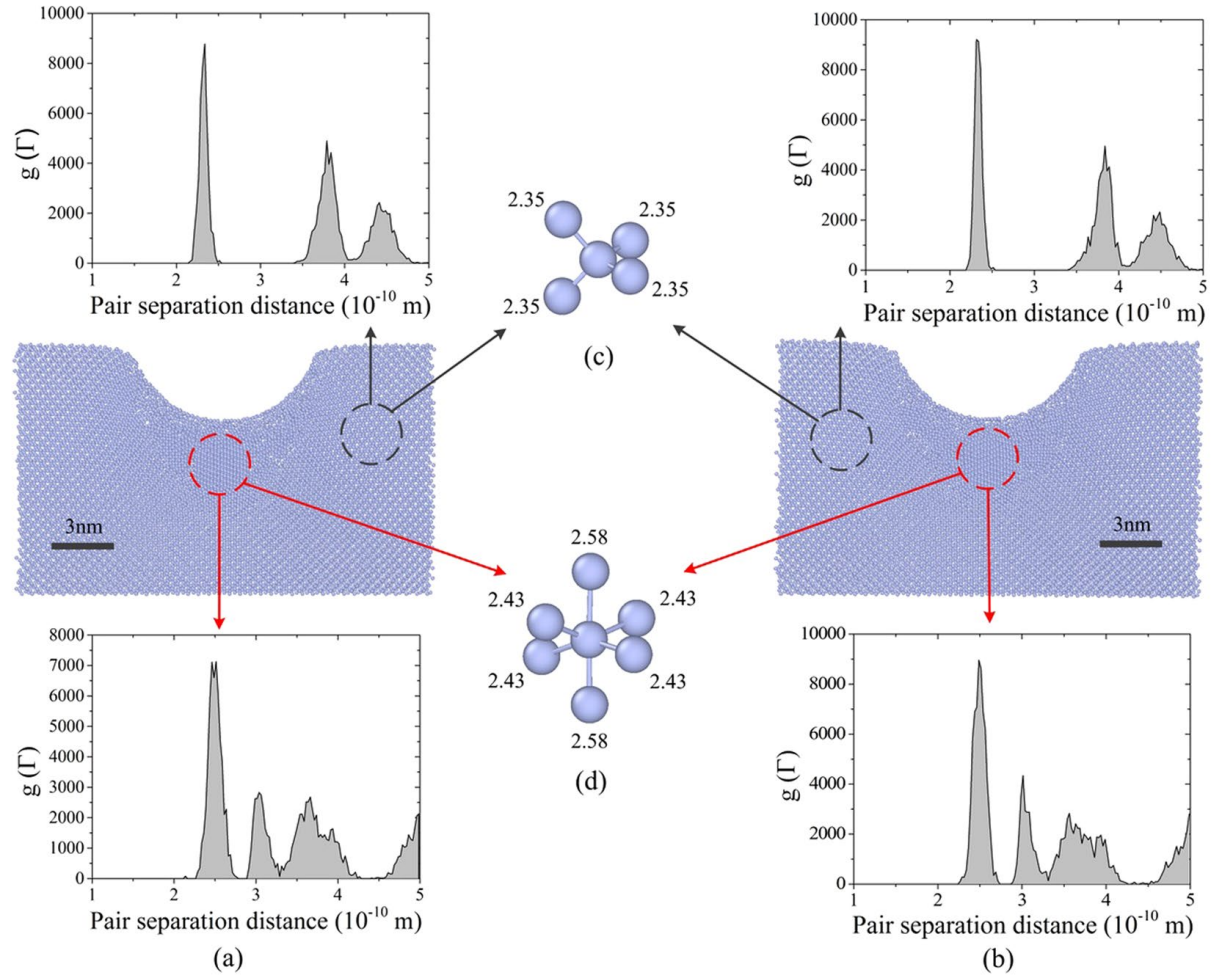
MD simulation of nanoindentation at 210 K and 292 K. (**a**) 210 K (**b**) 292 K. (**c**) The nearest atoms extracted from non-deformation region. (**d**) The nearest atoms extracted from the region under indenter.

To investigate the details of phase transformation during loading process, we perform MD simulations of nanoindentation on monocrystalline silicon. Figure 5a and b show the cross-sectional views of the constructed three dimensional models at the maximum depth of indentation at 210 K and 292 K respectively. A spherical tip was used to produce a relative homogeneous stress state of deformed silicon on the scale of nanometers. Generally, the Si-I phase exhibits a diamond cubic structure with each atom having four nearest neighbours at a distance of 2.35 Å, while Si-II phase has 4 nearest neighbours at a distance of 2.43 Å and 2 nearest neighbours at a distance of 2.58 Å^22–24^. At both temperatures, atoms with nearest neighbours from selected regions are extracted and shown in Fig. 5c and d. It is significant that the atoms at non-deformation region (marked with black circle) exhibit the phase state of Si-I, while the atoms just beneath the indenter (marked with red circle) perform as Si-II. The phase state is not influenced by temperature, which is consistent with the findings by Zhao *et al*.^12^. To determine the overall phase state of silicon, two regions are classified using radial distribution function (RDF), as shown in Fig. 5a and b. It can be seen that the RDF of two temperatures are also similar. The pair separation distance of the atoms at non-deformation region concentrates on the range between 2.19 Å and 2.50 Å, exhibiting the phase state of Si-I. At the region just beneath the indenter the pair separation distance concentrates on the range between 2.31 Å and 2.70 Å, which is superposed by the two kinds of nearest vibrating atoms of Si-II. From the MD simulation, it can be obtained that at the maximum depth of indentation, both phase states and phase distributions are similar at 210 K and 292 K, indicating that temperature has little effect on the phase transformation during nanoindentation loading process.

After confirming the Si-I has the ability transforming to Si-II, now we can further discuss the phase transformation process during unloading process of nanoindentation. According to the experimental results of Huang *et al*.^4^ and N. Fujisawa *et al*.^25^, the occurrence of pop-out at room temperature is due to the Si-III/XII phase reaching a critical volume as transformation seeds, and then grow up in a quit short period of time. The reduced probability of pop-out shown in Fig. 2 indicates that the generation of the transformation seeds is inhibited by the low temperature. With the temperature decreasing, Si-I becomes more and more difficult to transform to the initial Si-II. According to the decreasing trend of critical load for pop-out occurrence at each temperature, it can be assumed that because of the lower temperature, the generation of Si-III/XII needs a much more relax pressure condition. So temperature and pressure are two conditions controlling the phase transformation in silicon during unloading process. Additionally, the inhibition on phase transformation is also confirmed by the difference of *h-t* curves of pop-out occurred at 240 K and 292 K, as shown in Fig. 3. The pop-out occurred at 240 K takes a longer time and is smoother at the initial stage than the one at 292 K. The difference indicates that though the transformation seeds have existed because of the occurrence of pop-outs, the propagation of phase transformation from Si-II to Si-III/XII is quite inhibited by the low temperature, leading to a longer period of time to finish the pop-out. So that both the generation and propagation of Si-III/XII transformed from Si-II are inhibited by the low temperature. The *P-h* curves and Raman spectra show that low temperature inhibits the generation of Si-III/XII phase and promotes the *a*-Si generating. Both the phase state (*a*-Si) and phenomenon during unloading process (elbow) at low temperature are similar with the condition of room temperature with a rapid unloading rate, but the physical origins of the two kinds of elbows are quite different. At room temperature the appearance of elbow is due to Si-II phase lacking of sufficient time to transform into Si-III/XII seeds, while at low temperature the condition inhibits the generation and propagation of Si-III and Si-XII.

In summary, nanoindentation study coupled with MD simulations reveals that at low temperature Si-II phase can be transformed from Si-I at high pressure. But both the generation and propagation of Si-III/XII are inhibited by the low temperature, and as a result Si-II phase can only transform to *a*-Si during the pressure releasing.

## Methods

### Sample information

The monocrystalline silicon (100) is provided by MTI Corporation in Hefei, China. The thickness of this sample is approximately 3 mm, and surface roughness of the polished side is less than 0.5 nm. This side is used for nanoindentation measurements.

### Apparatus and low temperature nanoindentation experiments

**Figure 6:**
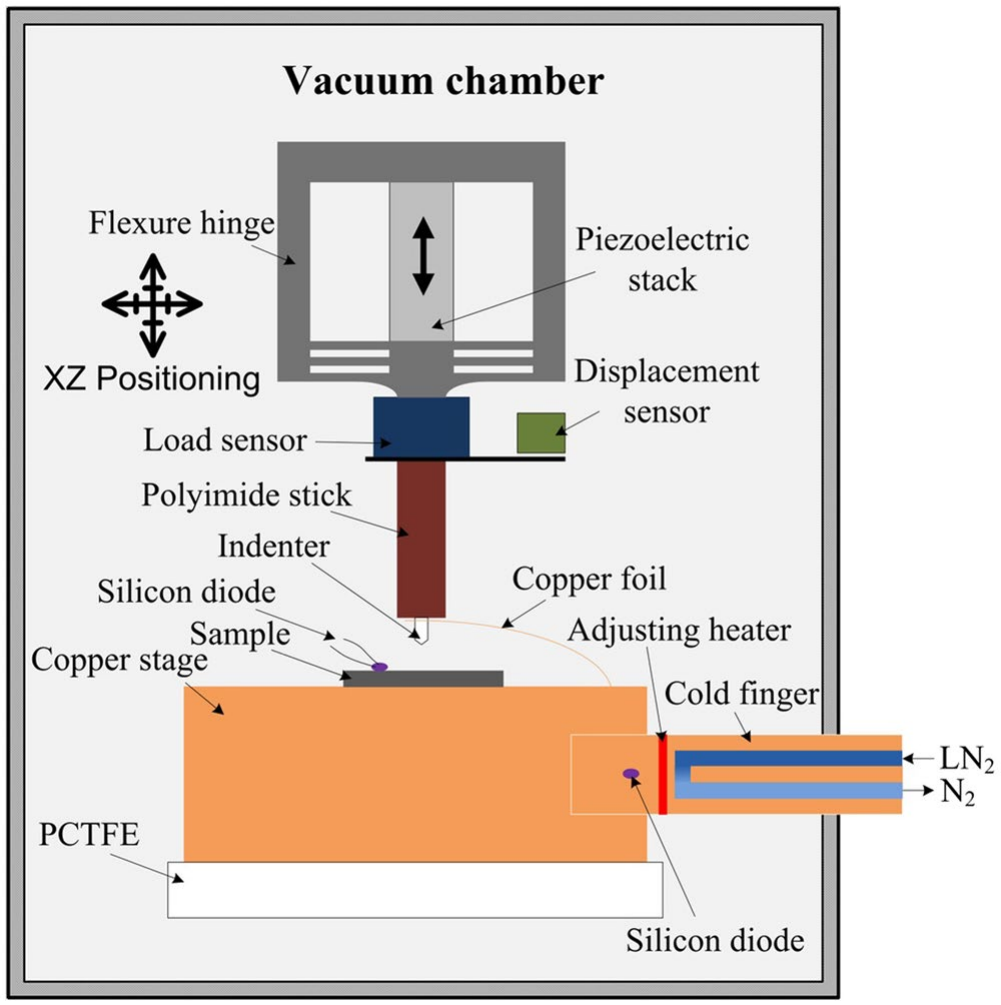
Schematic of the low temperature nanoindentation device.

Nanoindentation tests with a sapphire Berkovich indenter (Synton-MDP) were performed via a costumed cryogenic nanoindentation device as shown in Fig. 6. The details of the instrument without cryogenic function can be found in ref. 26. The cryogenic cooling system (Janis Company, ST-400) in current investigation used liquid nitrogen (LN_2_) to implement the cooling effect. LN_2_ absorbed heat with flowing through the cold finger, and then existed with a state of nitrogen (N_2_). The adjusting heater settled at the top of cold figure was used to control the final temperature of the copper stage by adjusting the output power. As the flow rate of LN_2_ and power of adjusting heater were both carefully controlled, finally the temperature fluctuation of copper stage could be controlled within ±0.1 K. Two silicon diodes (Cryo-con Company, S900-CP) were used, which one was settled inside the cold finger to complete the PID feedback control with adjusting heater, and another was settled on the surface to monitor the temperature of specimen, connected with thermally conductive grease (M&I Company, Apiezon N). A piece of copper foil was placed to connect the tip and the copper stage to cool down the tip. The tip was also forced to touch the specimen under a load of 500 mN for 60 min at each temperature to guarantee the temperature between tip and specimen is closed. Then 25 nanoindentation experiments were carried out with the maximum indentation load of 80 mN, and the load/unload rate was 1.6 mN/s. Meanwhile, all the indentation experiments were performed inside vacuum environment with a vacuum degree of 10^−2^ Pa to prevent the occurrence of ice.

### MD simulations

MD simulations were conducted to identify the existence of Si-II during low temperature nanoindentation. Periodic boundary conditions were chosen for the X and Z direction to reduce the effect of the simulation scale, and Y direction was built as assuming free boundary condition. Three-dimensional models were established and a thick of 4 Å atoms was selected to exhibit the cross section of nanoindentation. The MD model of the specimen was equilibrated to the target temperature of 292 K and 210 K respectively, assuming a microcanonical (NVE) ensemble^–^.
